# Early surgery may lower mortality in patients suffering from severe spinal infection

**DOI:** 10.1007/s00701-020-04507-2

**Published:** 2020-07-29

**Authors:** Sara Lener, Christoph Wipplinger, Anna Stocsits, Sebastian Hartmann, Anja Hofer, Claudius Thomé

**Affiliations:** Department of Neurosurgery, Medical University Innsbruck, Anichstrasse 35, 6020 Innsbruck, Austria

**Keywords:** Spinal infection, Spondylodiscitis, Mortality, Infection, Morbidity

## Abstract

**Purpose:**

Spinal infection (SI) is a life-threatening condition and treatment remains challenging. Numerous factors influence the outcome of SI and both conservative and operative care can be applied. As SI is associated with mortality rates between 2 and 20% even in developed countries, the purpose of the present study was to investigate the occurrence and causes of death in patients suffering from SI.

**Methods:**

A retrospective analysis was performed on 197 patients, categorized into two groups according to their outcome: D (death) and S (survival). The diagnosis was based on clinical and imaging (MRI) findings. Data collected included demographics, clinical characteristics, comorbidities, infection parameters, treatment details, outcomes, and causes of death.

**Results:**

The number of deaths was significantly higher in the conservative group (*n* = 9/51, 18%) compared with the operative counterpart (*n* = 8/146, 6%; *p* = 0.017). Death caused by septic multiorgan failure was the major cause of fatalities (*n* = 10/17, 59%) followed by death due to cardiopulmonary reasons (*n* = 4/17, 24%). The most frequent indication for conservative treatment in patients of group D included “highest perioperative risk” (*n* = 5/17, 29%).

**Conclusion:**

We could demonstrate a significantly higher mortality rate in patients solely receiving conservative treatment. Mortality is associated with number and type of comorbidities, but also tends to be correlated with primarily acquired infection. As causes of death are predominantly associated with a septic patient state or progression of disease, our data may call for an earlier and more aggressive treatment. Nevertheless, prospective clinical trials will be mandatory to better understand the pathogenesis and course of spinal infection, and to develop high quality, evidence-based treatment recommendations.

## Background

Spinal infection (SI) is a life-threatening condition and defined as an infectious disease affecting the spine and/or the paravertebral tissues [20]. SI may arise primarily by hematogenous spread, or secondarily as a result of spinal surgery or trauma [3, 29]. The incidence of SI has been increasing as a result of an aging population with serious comorbidities and the rising number of spinal interventions in the last few decades. Additionally, improved diagnostic capabilities have led to an earlier and more accurate diagnosis [6, 28]. To date, SI represents 2 to 7% of all musculoskeletal infections [27]. Early diagnosis of SI is challenging, and adequate treatment is complex, especially in older patients with comorbidities. Conservative treatment options are successful in many cases, but may not suffice. Surgery has to be considered when medical options have failed and symptoms or imaging findings progress. Especially immobilized older patients may benefit from early operative treatment to prevent complications of failed conservative treatment. Numerous factors influence the course and outcome of SI, including age, etiology, severity of comorbidities, as well as the implementation of conservative and operative treatment. Although several guidelines for the treatment of SI are available, therapy is not standardized and is mostly based on individual preferences and experience. SI is still associated with mortality rates between 2 and 20% in developed countries and is therefore considered a life-threatening condition [1, 24]. Thus, the purpose of the present retrospective study was to investigate the occurrence and causes of death in patients suffering from SI.

## Methods

We performed a retrospective analysis of all patients presented to our department with an SI between 2010 and 2017. Patients were categorized into two groups according to their outcome: D (death) and S (survival). The diagnosis was based on clinical magnetic resonance imaging (MRI) findings and changes in infection parameters. Data was collected using the patients’ digital health records (Cerner Millennium – Power Chart, Cerner Corporation 2011, Idstein, Germany) and was documented according to institutional standards and the general standards according to the principles of good clinical practice (GCP). Data included demographics, ASA scores, clinical and neurological characteristics measured by the muscle force assessment (0–5) according to the Medical Research Council (MRC), occurrence of comorbidities, age-adjusted Charlson comorbidity index (ACCI), infection parameters, such as C-reactive protein (CRP), treatment details and indications, respectively (operative vs. conservative), and treatment outcomes as well as causes of death. Sepsis was defined by the presence of two or more systemic inflammation response syndrome (SIRS) criteria. Patients suffering from (1) neurological deficits, (2) progressive or intractable pain, and/or (3) radiological progression due to MRI findings despite maximum conservative treatment qualified for surgical treatment, after being medically cleared for surgery by an interdisciplinary team. The operative procedure was determined individually for each patient, depending on present comorbidities, the extent and location of the infection, and the grade of vertebral destruction. All patients were additionally treated with intravenous broad-spectrum antibiotics (primarily clindamycin and ciprofloxacin, adjusted to the antibiogram whenever available) [12].

### Statistical analysis

All patients with complete initial data were considered for inclusion in the retrospective analysis. Values are expressed by mean ± standard deviation (SD). The Kolmogorov-Smirnov test was used for testing normal distribution. The unpaired Student’s *t* test and Mann-Whitney *U* test were performed to analyze differences in clinical and demographic characteristics and in clinical outcome variables. Frequencies were compared by the Chi-square and Fisher’s exact tests. Spearman’s rho correlation (r) was determined to assess the relationship between clinical outcome and demographics. A *p* value < 0.05 was considered statistically significant. All statistical evaluations were performed with SPSS version 21.0 (IBM Corp. Released 2012. IBM SPSS Statistics for Mac OS X, version 21.0, NY: IBM Corp.). Figures were designed using Microsoft Excel (version 15.36 for Mac OS X, Microsoft Corporation 2017, Redmond, USA).

## Results

### Baseline characteristics

One hundred ninety-seven patients with a sufficient data set were identified and evaluated retrospectively. Overall mortality in our cohort was 8.6% (*n* = 17/197). Demographic details and patients’ characteristics are outlined in Table 1. Twenty-two (11.2%) patients suffered from isolated discitis or isolated spondylitis, respectively. One hundred thirty-six patients (69.0%) were diagnosed with spondylodiscitis, whereof 64 (47.1%) presented with an accompanying spinal epidural abscess (SEA) and 48 (35.3%) with an additional paravertebral abscess. Moreover, 6.1% (*n* = 12/197) of patients showed an isolated SEA without affection of the discs or vertebral bodies, whereas 5 patients (2.5%) presented with an isolated paravertebral abscess.

**Table 1:** Demographic details

		Group D *n* = 17 *(8.6)*	Group S *n* = 180 *(91.4)*	
Age	In years *(SD)*	71.2 (± 9.6)	65.1 (± 12.4)	n.s.
Sex, *n (%)*	Male	9 (52.9)	122 (67.8)	n.s.
	Female	8 (47.1)	58 (32.2)	
BMI	In kg/m2 *(SD)*	29.8 (±13.1)	25.9 (±4.6)	n.s.
ASA score, *n (%)*	°1	0 (0.0)	16 (8.9)	*p* < 0.01
	°2	1 (5.9)	52 (28.9)	
	°3	12 (70.6)	102 (56.7)	
	°4	4 (23.5)	10 (5.6)	
Etiology, *n (%)*	Primary	14 (82.4)	113 (62.8)	n.s.
	Secondary	3 (17.6)	67 (37.2)	
Treatment, *n (%)*	Operative	8 (47.1)	138 (76.7)	*p* < 0.05
	Conservative	9 (52.9)	42 (23.3)	
Abscess, *n (%)*	Epidural	4 (23.5)	60 (33.3)	n.s.
	Paravertebral	4 (23.5)	44 (24.4)	
Location of infection, *n (%)*	Cervical	2 (11.8)	22 (12.2)	n.s.
	Thoracic	5 (29.4)	32 (17.8)	
	Lumbar	9 (52.9)	107 (59.4)	
	Cervical and thoracic	0 (0.0)	2 (1.1)	
	Cervical and lumbar	0 (0.0)	1 (0.6)	
	Thoracic and lumbar	1 (5.9)	16 (8.9)	
Total number of comorbidities, *(SD)*		2.4 (±1.4)	1.7 (±1.3)	*p* < 0.05
Type of comorbidities, *n (%)*	Depression	3 (17.6)	15 (8.3)	n.s.
	Renal failure	9 (52.9)	24 (13.3)	*p* < 0.01
	Diabetes	7 (41.2)	33 (18.3)	n.s.
	Heart disease	10 (58.8)	62 (34.4)	n.s.
	Vascular disease	9 (52.9)	71 (39.4)	n.s.
	Hepatopathy	5 (29.4)	30 (16.7)	n.s.
	Dental disease	0 (0.0)	8 (4.4)	n.s.
Cancerous disease, *n (%)*	Active disease	2 (11.8)	10 (5.6)	n.s.
	Status post	2 (11.8)	14 (7.8)	n.s.
Noxae, *n (%)*	Smoking	0 (0.0)	31 (17.2)	n.s.
	Alcohol abuse	1 (5.9)	13 (7.2)	n.s.
	Drug abuse	3 (17.6)	23 (12.8)	n.s.
Blood culture, *n (%)*	Positive	7 (41.2)	40 (22.2)	n.s.
	Staph. aureus	5 (29.4)	15 (8.3)	*p* < 0.01
	other	3 (17.7)	21 (11.6)	n.s.

(*n* population, *SD* standard deviation)

### Management

**Fig. 1:**
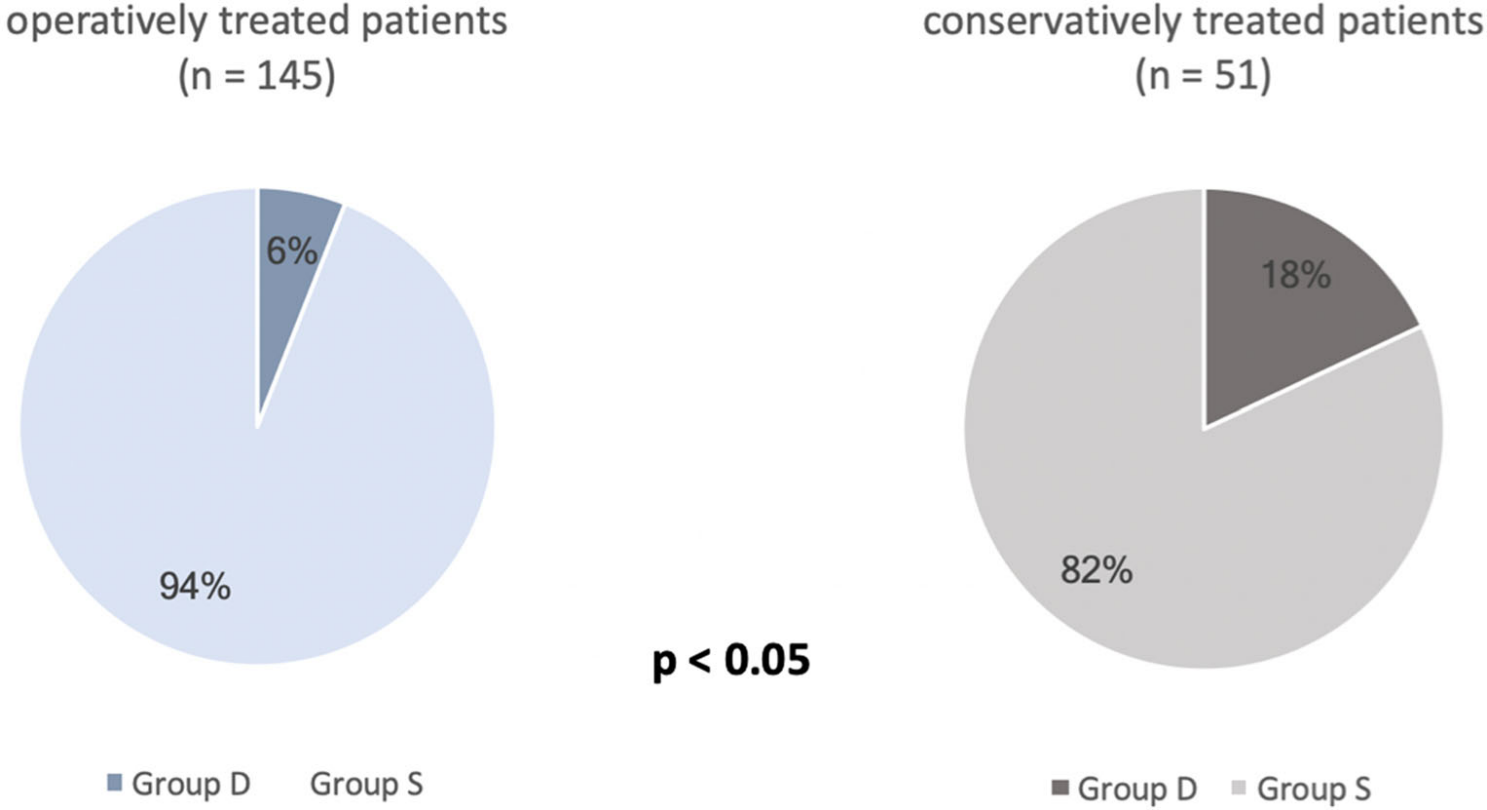
Mortality in patients treated operatively vs. conservatively. (n: population, D: death, S: survival)

Fifty-one patients (25.9%) were treated conservatively, and 146 patients (74.1%) were managed surgically. Depending on the extent of infection and bony destruction, surgical cases were only decompressed or decompressed and instrumented with or without (partial) corpectomy (Table 2). A CT-guided biopsy was performed initially in 35 patients (17.8%), and in 24 thereof (68.6%; 12.2% of the entire cohort), a pathogen could be detected. The most commonly isolated pathogen was multi-sensitive *Staphylococcus aureus* (*n* = 5/24, 20.8%). An antibiogram-adjusted antibiotic treatment due to a positive pathogen detection was conducted in 41.2% (*n* = 7/17) of patients in group D and in 28.8% (*n* = 52/180) of patients in group S (*p* > 0.05) (Fig. 1).

**Table 2:** Details of operative treatment for both groups

		Group D *n* = 8	Group S *n* = 138	
Operative time	In minutes *(SD)*	264 (± 108)	188 (± 89)	*p* < 0.05
Operation performed cervical, *n (%)*	Corpectomy and fusion	2 (25.0)	7 (5.1)	n.s.
	Fusion	1 (12.5)	28 (20.3)	
	Decompression	0	5 (3.6)	
Thoracic, *n (%)*	Corpectomy and fusion	3 (37.5)	4 (2.9)	
	Fusion	0	19 (13.8)	
	Decompression	0	0	
Lumbar, *n (%)*	Corpectomy and fusion	0	7 (5.1)	
	Fusion	2 (25.0)	66 (47.8)	
	Decompression	0	2 (1.4)	
Ventral approach, *n (%)*		3 (37.5)	25 (18.1)	n.s.
Dorsal approach, *n (%)*		5 (62.5)	113 (81.9)	

(*n* population, *SD* standard deviation)

### Causes of death

**Fig. 2:**
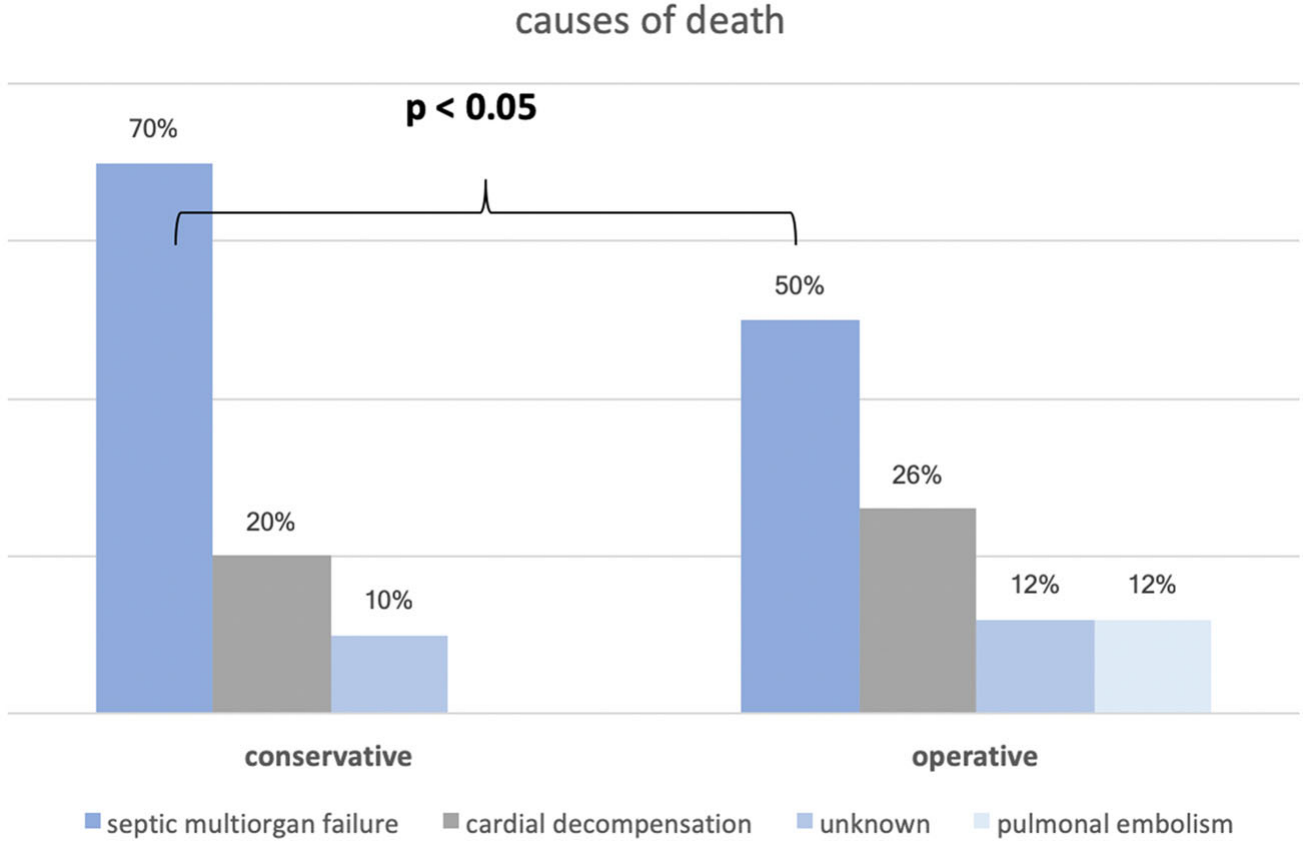
Causes of death in operatively vs. conservatively treated patients

The majority of patients (59%) died from septic multiorgan failure (*n* = 10/17), followed by death due to cardiopulmonary reasons (*n* = 4/17, 24%). Death by septic multiorgan failure was significantly more frequent in the conservatively treated group (*n* = 6/17; 35%) when compared with the surgically treated patients (*n* = 4/17; 24%; *p* < 0.05). The individual causes of death for both treatment groups are depicted in detail in Fig. 2. Reasons for the indication of a solely conservative treatment scheme in group D patients included “highest perioperative risk” (*n* = 5/17, 29%), “no neurological deficit” (*n* = 3/17, 18%), and “healing of abscesses before intervention” (*n* = 1/17, 6%). These indications justifying conservative treatment differed significantly between groups D and S (*p* ≤ 0.01). When rated as “highest perioperative risk” and treated conservatively, a patient’s chance to survive was 50% (*n* = 5/10). When treated conservatively due to missing neurologic symptoms, a survival rate of 92.3% was observed (*n* = 36/39). Indications for operatively treated patients in group D solely included “progression of symptoms” (*n* = 8/17, 47%). Individual treatment indications in both groups are shown in detail in Table 3.

**Table 3:** Individual treatment indications for both, conservative and operative treatment

	Group D	Group S
*Reasons for conservative treatment*		
Highest perioperative risk	29%	1%
No neurological deficit	18%	22%
Other prior treatment indicated	5%	1%
*Reasons for operative treatment*		
Progressive disease	18%	57%
Pain	12%	17%
Paraplegia	18%	2%

### Factors contributing to outcome

A higher mortality was observed in patients treated conservatively (*n* = 9/51, 18%) compared with surgically treated patients (*n* = 8/146, 6%; *p* = 0.017; Fig. 1). This was also the case in patients classified with ASA 3 or higher (conservatively treated: *n* = 8/32, 25% vs. operatively treated: *n* = 8/96, 8%; *p* < 0.01). Overall, patients in group S demonstrated a significantly lower ASA score (mean: 2.6 ± 0.7) when compared with group D (mean: 3.2 ± 0.5; *p* ≤ 0.01). Duration of surgery differed significantly between group S (188 ± 89 min) and group D (264 ± 108 min; *p* = 0.031). The overall time interval from diagnosis to death in group D was 110 ± 294 days, whereas the time from surgery to death was 43 ± 60 days.

The occurrence of an epidural or a paravertebral abscess was not associated with higher mortality (*p* > 0.05). The total number of accompanying illnesses per patient differed significantly between both groups (S: 1.7 ± 1.3 vs. D: 2.4 ± 1.4; *p* = 0.019). The ACCI also demonstrated significantly worse scores in group D (6.5 ± 2.3) than in group S (3.8 ± 2.3; *p* < 0.01). A higher mortality was shown for patients suffering from renal failure (S: *n* = 24/180, 13% vs. D: *n* = 9/17, 53%; *p* ≤ 0.01) and diabetes mellitus (DM) tended to be associated with a higher mortality rate as well (*p* = 0.071). Group D largely consisted of patients affected by primary acquired spondylodiscitis (*n* = 14/17, 82.4%) compared with group S (*n* = 113/180, 62.8%) (*p* > 0.05). Group D showed significantly higher CRP levels (D: 23.1 ± 12.9 mg/dl vs. S: 10.9 ± 11.1 mg/dl; *p* = 0.01) and signs of sepsis at admission (D: *n* = 13/17, 76.5% vs. S: *n* = 38/180, 21.1%; *p* ≤ 0.01). Also, patients in group D were admitted with significantly more pronounced neurological deficits, measured by the weakest muscle group according to the MRC (S: 3.9 ± 1.5 vs. D: 1.7 ± 1.8; *p* = 0.002). In group S, neurological deficits as well as infection parameters, improved significantly during the hospital stay (*p* < 0.05) whereas in group D parameters tended to improve over time, but this failed to reach statistical significance (*p* > 0.05).

## Discussion

We report the results of a retrospective analysis investigating the relevant factors for mortality in spinal infections. Deceased patients were more frequently affected by primary acquired spondylodiscitis, and patients died significantly more often under conservative treatment compared with the surgical counterpart. Two thirds of patients died from septic multiorgan failure followed by death due to cardiopulmonary reasons. Indications for solely conservative treatment in patients who died included “highest perioperative risk,” “no neurological deficit,” and “prior healing of abscesses,” whereas reasons for surgical intervention were limited to “progression” for this particular patient group.

The course of primarily acquired SI may be more severe than that of postoperatively acquired SI [29]. A higher mortality of primarily acquired SI was observed in our retrospective analysis; however, it failed to reach statistical significance. Our data did not show any correlation of a para/epidural abscess with mortality. Still, primarily acquired spondylodiscitis was more likely to be associated with a paravertebral or an intraspinal abscess [29]. Previous data revealed that the higher mortality and a more severe course of primarily arisen SI may be linked to a more frequent occurrence of abscesses, which could not be demonstrated in our cohort. A preexisting compromised immune status of affected patients would be plausible as other underlying reason for the serious course of primarily acquired SI.

It is widely known that renal failure is associated with a higher mortality in infection and septic shock [16, 26]. Furthermore, it may not only show validity in primary infection but also in postoperatively occurring infections. Mortality in secondary acquired SI was shown to be nearly three times higher when accompanied by chronic renal failure [18, 21]. Underlying reasons include that kidney failure and uremia are associated with severe alterations of the immune system, as the mechanisms required for complete activation of T cells are compromised [8, 22, 30]. Moreover, in chronic kidney disease, the neutrophil count may not be altered, but neutrophils are less capable to eradicate microorganisms [17]. The occurrence of DM showed a trend to be correlated with higher mortality. In our cohort, however, this finding failed to reach statistical significance. Nevertheless, special attention should be paid to patients suffering from DM and presenting with SI, as DM is a known risk factor for surgical site infection as well as for the development of septic shock. Thus, patients with DM and an HbA1c above 7.5% are not scheduled for elective spine surgery at the authors’ department. This goes in line with the literature threshold of Hba1c ≥ 7.0% [5, 9, 14]. Overall, the total number of patients’ comorbidities and a subsequently higher ASA score seemed to be a predictive factor for increased mortality when affected by an SI. Several measurements to evaluate the impact of comorbidities and the estimation of morbidity and mortality have been designed to evaluate and grade the degree of comorbidity burden, one of them being the ACCI [4]. It is known to be a reliable parameter to predict mortality in various disease patterns and especially in bacteremia [15]. It has been reported that Charlson score > 3 to > 6 is predictive for developing severe sepsis, both postoperatively and primarily acquired [10, 13, 19]. A similar correlation could be shown in previous studies, in which a preoperative ASA > 2 led to significantly increased death rates in patients treated with spinal instrumentation [24]. Compared with our cohort, this may also be true for patients being treated for SI.

Treatment strategies for SI still remain controversial and the implementation of the optimal therapy for each patient needs to be individualized. Conservative cases seem to be followed by mechanical low back pain more often than surgical cases and develop more deformity in the long term. This appears particularly true for cervical cases, in which an infectious kyphosis often represents the final stage of SI [23]. To overcome this drawback by surgery comes at a price, as complication rates are higher in surgically treated cases. Overall mortality, however, is lower in operated patients [28]. Even multimorbid patients at an advanced age may show better overall outcomes when treated surgically, despite an increased risk of perioperative complications. It has been shown that delayed surgical treatment entails significantly poorer surgical outcome [2, 7]. In their analysis of 34,465 patients, Segreto et al. clearly demonstrated that delayed surgery is associated with increased mortality and complication rates [25]. Thus, it has been favored to indicate early surgery even in sick and comorbid patients.

The overall mortality in our cohort was 8.6% and is comparable with recently reported rates between 2 and 20% in developed countries [1, 24]. Patients died significantly more often when treated conservatively, which substantiates the aforementioned opinions. If patients were not considered appropriate candidates for surgery due to “highest perioperative risk,” i.e., they presented in a poor general clinical condition, they had a 50% risk to die. According to the available literature, this may well surpass the perioperative risk, particularly if treated early after onset of symptoms. Therefore, when it comes to decide whether a conservative or an operative strategy should be performed, the potential risk of surgery outweighing the risk of death due to a surgically untreated septic shock has to be taken into strong consideration. Furthermore, the whole team including anesthesia should be aware of significantly higher mortality rates in patients not operated, especially when scored with ASA 3 or higher. In particular, septic multiorgan dysfunction was the most frequently reported reason for death in our cohort, even significantly more common in the conservatively treated patient group. The second most frequent cause of death was cardiopulmonary issues (i.e., cardiac decompensation, respiratory insufficiency, pulmonary embolism) which may also be related to the septic status of the patients. As a result, this leaves room for discussion if the focus on the infection, which in those cases with great probability caused death by septic multiorgan dysfunction, should at least potentially be extracted. This subsequently increases the patients’ chance of survival as it aims to remove the primary focus of infection.

The indications for medical treatment in group D differed significantly compared with the surviving patients cohort, with the surviving majority of patients mostly being treated conservatively because of a missing neurological deficit, but not due to the level of systemic inflammatory symptoms. Furthermore, surgically treated patients only died if the indication for surgery was “progression of symptoms.” Patients were treated medically until the disease was clinically and radiologically progressive and surgery had to be performed urgently. As mentioned before, delayed surgical treatment is already known to lead to a significantly poorer outcome in patients with SI [2, 7].

When managed surgically, patients who suffered death showed significantly longer operation times, consistent with a higher number of fused levels, as infection may have broadly spread. It is known that longer operative time might be associated with a higher mortality [11, 24]. Nevertheless, in our cohort, it is presumed that the operative time may play a subordinate role, and that the extension and severity of infection may be decisive for the patients’ outcome. Additionally, patients at a higher risk for surgical treatment with progressive SI might benefit from a reduced length of operation.

## Limitations

The main limitation of our study is its retrospective nature. There obviously is selection bias, as many SI patients do not even reach a supraregional referral center and are managed successfully with conservative care. Nevertheless, if patients fail conservative care, show progressive symptoms, or develop neurologic deficits, they will be transferred to our university hospital. Thus, our results are representative for this more severely affected subpopulation of SI patients.

Additionally, indication of surgery may vary according to the attending neurosurgeon, anesthesiologic triage, patient will, and expectations. Overall, however, literature data as well as our results suggest that delayed surgery increases mortality. Special attention should be paid on quickly identifying and managing comorbidities (i.e., renal failure, diabetes mellitus), especially in multimorbid patients. Although further prospective studies are mandatory, early surgery seems to be beneficial in severely affected SI patients.

## Conclusion

Spinal infection is a serious and life-threatening disease requiring urgent treatment. Our retrospective analysis demonstrates a significantly higher mortality rate in patients receiving solely conservative treatment. Mortality is frequently associated with number and type of comorbidities, but also trends to be correlated with primarily acquired infection. As causes of death are predominantly associated with a septic patient state or progression of disease, our data may call for an earlier and more aggressive treatment. Nevertheless, prospective clinical trials will be mandatory to better understand the pathogenesis and course of spinal infection and to develop high quality, evidence-based treatment recommendations.

## Abbreviations

ASA: American Society of Anesthesiologists
CRP: C-reactive protein
D: Death
DM: Diabetes mellitus
GCP: Good clinical practice
MRI: Magnetic resonance imaging
S: Survival
SD: Standard deviation
SI: Spinal infection
